# Supplementary material for: Content-rich biological network constructed by mining PubMed abstracts
Source: BMC Bioinformatics. 2004 Oct 8;5:147. doi: 10.1186/1471-2105-5-147 (PMC528731; doi:10.1186/1471-2105-5-147)
Supplement: Additional File 2 — The original results of the above study (non-essential files are deleted to keep the file size under the limit set by BMC bioinformatics). [file 1471-2105-5-147-S2.bz2 › chilibotAdditionalFile2/dip05/634ID10669618E11021/html/NDOB_NDOC.html]

 


 **NDOB** and **NDOC** 
  
Found 1 abstracts in PubMed, retrieved 1.  
 

 What does Google say? 
 PDF only 
| .edu only 

---

**Interactive relationship** (e.g. stimulation, inhibition, etc)

**Non-interactive relationship** (e.g. studied together, co-existance, homology, etc.)

- The NH2 terminal amino acid sequences of the alpha and beta subunits were identical to the deduced amino acid sequences reported for the  **ndoB**  and  **ndoC**  genes from P. putida NCIB 9816 and almost identical to the NH2 terminal amino acid sequences determined for the large and small subunits of ISPNAP from P. putida G7.  Ref: 8376335 J Bacteriol, 1993
